# Response to 2009 Pandemic Influenza A (H1N1) Vaccine in HIV-Infected Patients and the Influence of Prior Seasonal Influenza Vaccination

**DOI:** 10.1371/journal.pone.0016496

**Published:** 2011-01-31

**Authors:** Darius Soonawala, Guus F. Rimmelzwaan, Luc B. S. Gelinck, Leo G. Visser, Frank P. Kroon

**Affiliations:** 1 Department of Infectious Diseases, Leiden University Medical Center, Leiden, The Netherlands; 2 Department of Virology, Erasmus Medical Center, Rotterdam, The Netherlands; 3 Department of Internal Medicine, MC Haaglanden, the Hague, The Netherlands; University of California San Francisco, United States of America

## Abstract

**Background:**

The immunogenicity of 2009 pandemic influenza A(H1N1) (pH1N1) vaccines and the effect of previous influenza vaccination is a matter of current interest and debate. We measured the immune response to pH1N1 vaccine in HIV-infected patients and in healthy controls. In addition we tested whether recent vaccination with seasonal trivalent inactivated vaccine (TIV) induced cross-reactive antibodies to pH1N1. (clinicaltrials.gov Identifier:NCT01066169)

**Methods and Findings:**

In this single-center prospective cohort study MF59-adjuvanted pH1N1 vaccine (Focetria®, Novartis) was administered twice to 58 adult HIV-infected patients and 44 healthy controls in November 2009 (day 0 and day 21). Antibody responses were measured at baseline, day 21 and day 56 with hemagglutination-inhibition (HI) assay. The seroprotection rate (defined as HI titers ≥1∶40) for HIV-infected patients was 88% after the first and 91% after the second vaccination. These rates were comparable to those in healthy controls. Post-vaccination GMT, a sensitive marker of the immune competence of a group, was lower in HIV-infected patients. We found a high seroprotection rate at baseline (31%). Seroprotective titers at baseline were much more common in those who had received 2009–2010 seasonal TIV three weeks prior to the first dose of pH1N1 vaccine. Using stored serum samples of 51 HIV-infected participants we measured the pH1N1 specific response to 2009–2010 seasonal TIV. The seroprotection rate to pH1N1 increased from 22% to 49% after vaccination with 2009–2010 seasonal TIV. Seasonal TIV induced higher levels of antibodies to pH1N1 in older than in younger subjects.

**Conclusion:**

In HIV-infected patients on combination antiretroviral therapy, with a median CD4+ T-lymphocyte count above 500 cells/mm^3^, one dose of MF59-adjuvanted pH1N1 vaccine induced a high seroprotection rate comparable to that in healthy controls. A second dose had a modest additional effect. Furthermore, seasonal TIV induced cross-reactive antibodies to pH1N1 and this effect was more pronounced in older subjects.

## Introduction

Most guidelines recommend annual influenza vaccination of all HIV-infected patients [1]. The rationale for this recommendation is that in the era of widespread use of combination antiretroviral therapy (cART) influenza is still associated with increased rates of morbidity in HIV-infected patients [2], [3] and that vaccination prevents disease [4], [5]. The immunogenicity of adjuvanted 2009 pandemic influenza A(H1N1) (pH1N1) vaccines in HIV-infected patients and the effect of recent and past trivalent inactivated influenza vaccines (TIV) is a matter of current interest. We measured the humoral immune response to a monovalent MF59-adjuvanted surface-antigen vaccine containing 7,5 µg hemagglutinin of strain A/California/7/2009 (H1N1) (X-181) (Focetria®, Novartis) in HIV-infected patients and in healthy controls. In addition we tested whether recent vaccination with seasonal TIV induced cross-reactive antibodies to pH1N1.

## Methods

### Ethics statement

This study was approved by the ethics committee of Leiden University Medical Center (protocol number 09.187). Subjects provided written informed consent for participation in the study and for the use of stored serum samples for the purpose of this study.

### Study design and source population

This was a single-center prospective cohort study at Leiden University Medical Center in The Netherlands. The pH1N1 vaccine was administered twice to 58 adult HIV-infected patients (patients) and 44 healthy hospital employees (controls) in November and December 2009 (day 0 and day 21). Exclusion criteria were: use of systemic immunosuppressive medication, ongoing febrile illness, pregnancy or laboratory confirmed pH1N1 influenza before the first vaccination. At inclusion, participants were asked whether they had experienced symptoms of influenza in the two preceding months. In addition, all participants filled out a standardized diary on symptoms of influenza during the 56 day follow-up period. Influenza-like illness was defined as sudden onset of fever of >38°C and cough or sore throat in the absence of other diagnoses [6]. Serum was collected at baseline, at day 21 (just before the second dose) and at day 56 (35 days after the second dose). In a subset of 51 participants (29 patients and 22 controls) serum was also collected at day 7. We retrieved stored serum samples of a subset of 51 HIV-infected patients who had been vaccinated with unadjuvanted 2009–2010 seasonal trivalent inactivated influenza vaccine (TIV) a month before receiving the first pH1N1 vaccination. In addition, we retrieved stored samples of 14 of these 51 HIV-infected patients who had also participated in an influenza vaccination trial in 2005 [7]. There were no such samples available of the healthy controls. The stored serum samples were used to measure whether 2009–2010 and 2005–2006 seasonal TIV induced cross-reactive antibodies to pH1N1 influenza.

### Laboratory analysis and main outcome measures

Antibodies to the vaccine strain A/California/7/2009 (H1N1) and to the seasonal influenza vaccine strains A/NewCaledonia/20/1999 and A/Brisbane/59/2007 were measured using the hemagglutination-inhibition (HI) assay, according to standard methods [8]. Titers below the detection limit (i.e. <1∶10) were assigned a value of 1∶5. Geometric mean titers (GMTs) and seroprotection rates (defined as HI titers ≥1∶40) were the main outcome measures. Seroconversion was defined by a post-vaccination HI titer of at least 1∶40 combined with at least a four-fold increase in titer in accordance to European and international guidance [9], [10].

### Statistical methods

The between group difference in GMT taken over the three time points (day 0, 21, 56) was analyzed using a mixed linear model. This model takes into account that each subject had repeated measurements of the HI titer over time. We analyzed which variables predicted the level of post-vaccination GMT in the group of HIV-infected patients using a linear regression model with step-wise introduction of the continuous variables ‘log of the HI titer at baseline’, ‘age in years’, ‘CD4+ T-lymphocyte count (cells/mm^3^)’, ‘nadir CD4+ T-lymphocyte count (cells/mm^3^)’ and the categorical variables ‘HIV-1 RNA’ (<20 copy/ml, 20–400 copy/ml, >400 copy/ml) and ‘gender’. Proportions were compared with Pearson χ^2^ or Fisher's exact tests as appropriate. We explored which variables were associated with a baseline HI titer of ≥1∶40 using a logistic regression model by step-wise introduction of the continuous variable ‘age’ and the categorical variables ‘HIV-status’ (i.e. infected or healthy control), ‘gender’, ‘an influenza-like illness prior to inclusion’, ‘vaccination with 2009–2010 seasonal influenza vaccine’, ‘vaccination with 2008–2009 seasonal influenza vaccine’ and ‘vaccination with 2007–2008 seasonal influenza vaccine’.

In an exploratory analysis we looked at the effect of age on the level of cross-reactive antibodies to pH1N1 following 2009–2010 seasonal TIV using a linear regression model with step-wise introduction of the continuous variables ‘age in years’, ‘CD4+ T-lymphocyte count (cells/mm^3^)’, ‘nadir CD4+ T-lymphocyte count (cells/mm^3^)’ and the categorical variable ‘HIV-1 RNA’. This analysis was restricted to HIV-infected patients who had received seasonal TIV before pH1N1 vaccine and who had no measurable HI titer to pH1N1 prior to receiving 2009–2010 seasonal TIV.

## Results

Follow-up was complete for 98% (57/58) of HIV-infected patients and all healthy controls. The mean age of the patients was 52 (SD 11) years and of the controls 49 (SD 10) years. Of the patients, 91% (52/57) was on cART of whom 87% (45/52) had undetectable plasma HIV-1 RNA (<20 copies/mL) at baseline. The median CD4+ T-lymphocyte count was 507 (IQR 349-697) cells/mm^3^ and only three patients had a count below 200 cells/mm^3^. In the month preceding inclusion, 89% (51/57) of HIV-infected patients and 64% (28/44) of controls had been vaccinated with non-adjuvanted 2009-2010 seasonal TIV (Table 1).

**Table 1:** Participant Demographics.

	HIV-infected*n* = 57	Healthy Control*n* = 44
Male - *n* (%)	48 (84)	27 (61)
Age, years - *mean* (SD)	52 (11)	49 (10)
Age categories - *n* (%)		
18–44 years	14 (25)	12 (27)
45–59 years	26 (46)	28 (64)
>60 years	17 (30)	4 (9)
combination antiretroviral therapy (cART) - *n* (%)	52 (91)	-
baseline value CD4+ T-lymphocytes, cells/mm^3^ - *median* (IQR)	507 (349–697)	-
CD4 category, at the time of vaccination *n* (%)		-
<350 cells/mm^3^	14 (25)	-
>350 cells/mm^3^	43 (75)	-
nadir CD4+ T-lymphocytes, cells/mm^3^ - *median* (IQR)	143 (32–281)	-
baseline HIV-1 RNA - *n* (%)		-
<20 copy/ml	45 (79)	-
20-400 copy/ml	7 (12)	-
>400 copy/ml	5 (9)	-
past seasonal trivalent inactivated influenza vaccination - *n* (%)		
2009–2010	51 (89)	28 (64)
2008–2009	50 (88)	27 (61)
2007–2008	45 (79)	29 (66)

**Figure 1:**
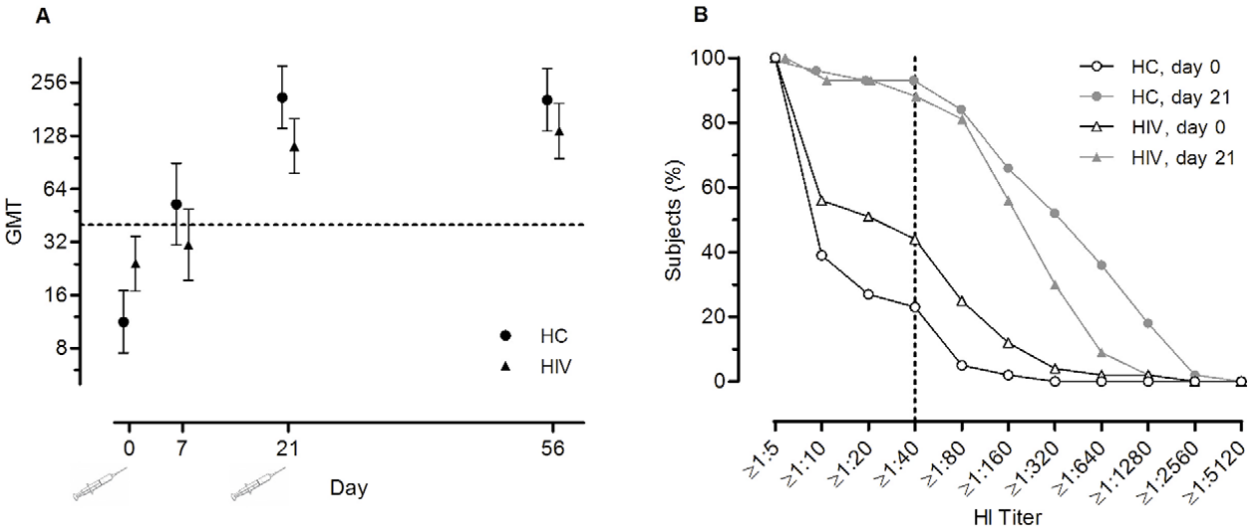
Immunogenicity of two doses of pH1N1 vaccine. Monovalent MF59-adjuvanted pandemic influenza vaccine (A/California/7/2009) administered to a group of 57 HIV-infected patients (HIV) and 44 healthy controls (HC). The vaccine was administered at day 0 (baseline) and at day 21. Age adjusted geometric mean titers with 95% confidence intervals at baseline, day 7, day 21 and day 56 (Panel A). Reverse cumulative distribution curves on hemagglutination inhibition assay at baseline and at day 21 (Panel B).

Three patients (5%) and 3 controls (7%) reported an influenza-like illness in the two months preceding inclusion, of whom 2 patients and 1 control had a baseline HI titer ≥1∶40. The baseline GMT was higher in patients (23, 95% CI 15–35) than in controls (12, 95% CI 8–16) (Figure 1a). At baseline, 44% (25/57) of patients and 23% (10/44) of controls had a HI titer ≥1∶40. Titers above 80 were uncommon at baseline (Figure 1b).

Immunogenicity results are summarized in Figure 1 and Table 2. In a mixed linear model, the age-adjusted average GMT taken over the three time points after vaccination was a factor 1.6 higher in controls than in HIV-infected patients (95% CI 1.0-2.5, *p* = 0.06) (Figure 1a). In a linear regression model restricted to the HIV-infected patients, only higher baseline titers (*p* = 0.02) were associated with higher HI titers at day 21. This association was not seen at day 56.

**Table 2:** Humoral immune response to two doses of pH1N1 vaccine.

	HIV-infected *n* = 57			Healthy Control *n* = 44			
	prior 2009-2010 seasonal TIV			prior 2009-2010 seasonal TIV			
	Yes*n* = 51	No*n* = 6	All*n* = 57	Yes*n* = 28		No*n* = 16	All*n* = 44
**pre-baseline, before 2009-2010 seasonal TIV (day –95)** *							
HI titer ≥1∶40 - *n* (%)	11 (22)	-	-		-	-	-
GMT – value (95% CI)	9(7–12)	-	-		-	-	-
**baseline, after 2009–2010 seasonal TIV but before 1^st^ pH1N1 vaccine (day 0)**							
HI titer ≥1∶40 - *n* (%)	25 (49)	0	25 (44)		9 (32)	1 (6)	10 (23)
GMT – value (95% CI)	28(18–42)	5 (-)	23(15–35)		15(9–25)	7(5–9)	12(8–16)
**after 1^st^ pH1N1 vaccine (day 21)**							
HI titer ≥1∶40 - *n* (%)	47 (92)	3 (50)	50 (88)		25 (89)	16 (100)	41 (93)
seroconversion – *n* (%)#	27 (53)	3 (50)	30 (53)		16 (57)	16 (100)	32 (73)
GMT – value (95% CI)	119(87–163)	57(16–193)	110(81–150)		117(69–198)	632(422–947)	216(139–334)
**after 2^nd^ pH1N1 vaccine (day 56)**							
HI titer ≥1∶40 - *n* (%)	47 (92)	5 (83)	52 (91)		23 (82)	16 (100)	39 (89)
seroconversion – *n* (%)#	31 (61)	5 (83)	36 (63)		15 (54)	16 (100)	31 (70)
GMT – value (95% CI)	138(101–187)	107(58–200)	134(101–178)		117(73–186)	572(384–853)	208(140–310)

Seroprotection- and seroconversion rates and geometric mean titers (GMT) to 2009 pandemic influenza A(H1N1) (pH1N1) virus for 57 HIV infected individuals and 44 healthy controls following vaccination with two doses of monovalent MF59-adjuvanted pandemic influenza vaccine (A/California/7/2009). Results are stratified by whether or not participants had been vaccinated with 2009–2010 seasonal trivalent inactivated influenza vaccine (TIV) before receiving the first pH1N1 vaccine.

*For 51 HIV-infected participants who had already been vaccinated with 2009–2010 seasonal TIV at baseline (day 0), we also determined HI titers to 2009 pandemic influenza A(H1N1) virus in stored serum samples that had been collected before they received 2009–2010 seasonal TIV.

# Baseline titers (day 0) were used as denominaters to determine seroconversion rates.

The seroprotection rate, defined as a titer ≥1∶40, was 88% (50/57) for HIV-infected patients three weeks after the first pH1N1 vaccination and 91% (52/57) after the second vaccination. For controls this was 93% (41/44) and 89% (39/44) respectively (Figure 1b). In a separate analysis, restricted to participants with a baseline titer below the detection limit, the seroprotection rate was 72% (18/25) for HIV-infected patients after the first and 88% (22/25) after the second vaccination. For the controls this was 89% (24/27) and 85% (23/27).

After the first vaccination only 53% (30/57) of HIV-infected patients achieved seroconversion compared with 73% (32/44) of controls. After the second vaccination this was 63% (36/57) and 70% (31/44) (Table 2). The GMT was lower in those who did not seroconvert than in those who did. The GMT in HIV-infected patients who did not seroconvert was 72 (95% CI 42–124) and was 161 (95% CI 122–212) in those who did seroconvert. For controls this was 61 (95% CI 25–147) and 347 (95% CI 233–516). As is to be expected, seroconversion rates were lower in those with high HI titers at baseline. In a separate analysis of 25 HIV-infected patients who had HI titers below the detection limit at baseline, 72% (18/25) achieved seroconversion after the first pH1N1 vaccination and 88% (22/25) after the second vaccination. For the 27 controls this was 89% (24/27) and 85% (23/27).

After the first vaccination, between day 0 and day 21, an influenza-like illness was reported by 5 HIV-infected patients (9%) and 6 controls (14%). Of these participants, 4/5 patients (80%) and 6/6 controls (100%) had a HI titer ≥1∶40 at day 21. In addition, one patient and 1 control reported an influenza-like illness between day 21 and day 56 of follow-up. Both had HI titer ≥1∶40 at day 56.

There were no serious adverse events following vaccination and HIV-1 RNA remained below the detection threshold in a random selection of 20 patients with undetectable viral loads at baseline.

All except 1 of the 35 subjects with a baseline pH1N1 titer ≥1∶40 had received 2009–2010 seasonal TIV. Prior vaccination with 2009–2010 seasonal TIV (OR 14, 95% CI 2–113, p = 0.01) and higher age (OR 1.04, 95% CI 1.0–1.1 for an increase in age by 1 year, p = 0.05) were associated with a baseline pH1N1 HI titer ≥1∶40.

Using stored serum samples of 51 of the HIV-infected patients we measured the pH1N1 specific response to 2009–2010 seasonal TIV administered a median of 17 days (IQR 14–23 days) before the first pH1N1 vaccination. We found that the seroprotection rate to pH1N1 increased from 22% to 49% following vaccination with 2009–2010 seasonal TIV and that 31% seroconverted (Table 2). This effect was age dependent. In a regression analysis restricted to 40 HIV-infected patients who all had undetectable HI titers to pH1N1 prior to vaccination with 2009–2010 seasonal TIV, we found that 2009–2010 seasonal TIV induced higher HI titers in older than in younger subjects (HI titer increased by a factor 1.05 95% CI 1.01–1.08 for an increase in age by 1 year, p = 0.01). This effect was independent of the CD4+ T-lymphocyte count, nadir CD4+ T-lymphocyte count and HIV-1 RNA. Of note, we found no evidence indicating that the immune response to pH1N1 vaccine was augmented by prior vaccination with seasonal TIV.

In a subset of 14 HIV-infected patients we measured (cross-reactive) pH1N1 HI titers following three different influenza vaccinations (i.e. 2005–2006 seasonal TIV, 2009–2010 seasonal TIV and pH1N1 vaccine). In 2005 the seroprotection rate to pH1N1 for this cohort of 14 HIV-infected patients increased from 14% to 43% after vaccination with 2005–2006 seasonal TIV (Table 3). In 2009 the seroprotection rate to pH1N1 had dropped back to 7% but increased to 50% after vaccination with 2009–2010 seasonal TIV. The subjects who developed cross-reactive antibodies to pH1N1 after 2005–2006 seasonal TIV were not necessarily the same subjects who did so after 2009–2010 seasonal TIV (p = 0.5, Fisher's exact test for the association between seroconversion to pH1N1 following 2005–2006 seasonal TIV and 2009–2010 seasonal TIV).

**Table 3:** (Cross-reactive) antibody titers following two different seasonal influenza vaccines in a cohort of 14 HIV-infected patients.

	HIV-infected (*n* = 14)*	
**influenza strain used in HI assay**	A/NewCaledonia/20/1999 (seasonal strain)	A/California/7/2009 (pandemic strain)
**Before 2005–2006 seasonal TIV**		
HI titer ≥1∶40 - *n* (%)	7 (50)	2 (14)
GMT – value (95% CI)	39 (16–92)	10 (5–17)
**after 2005–2006 seasonal TIV**		
HI titer ≥1∶40 - *n* (%)	11 (79)	6 (43)
seroconversion - *n* (%)	4 (29)	3 (21)
GMT – value (95% CI)	118 (52–272)	21 (10–45)
**influenza strain used in HI assay**	A/Brisbane/59/2007 (seasonal strain)	A/California/7/2009 (pandemic strain)
**Before 2009–2010 seasonal TIV**		
HI titer ≥1∶40 - *n* (%)	12 (86)	1 (7)
GMT – value (95% CI)	55 (34–90)	6 (4–9)
**after 2009–2010 seasonal TIV but before 1^st^ pH1N1 vaccine**		
HI titer ≥1∶40 - *n* (%)	14 (100)	7 (50)
seroconversion - *n* (%)	2 (14)	6 (43)
GMT – value (95% CI)	103 (57–187)	23 (12–43)
**after 1^st^ pH1N1 vaccine**		
HI titer ≥1∶40 - *n* (%)	-	13 (93)
seroconversion - *n* (%)	-	8 (57)
GMT – value (95% CI)	-	114 (62–209)

Seroprotection- and seroconversion rates and geometric mean titers (GMT) to 2005–2006 seasonal influenza A(H1N1) virus, to 2009–2010 seasonal influenza A(H1N1) virus and to 2009 pandemic influenza A(H1N1) (pH1N1) virus for 14 HIV infected individuals following vaccination with seasonal trivalent inactivated influenza vaccine (TIV) in 2005 (A/New Caledonia/20/1999 (H1N1) like strain), with seasonal TIV in October 2009 (A/Brisbane/59/2007 (H1N1) like strain) and with a first dose of monovalent MF59-adjuvanted pH1N1 vaccine (A/California/7/2009) in November 2009.

*Population characteristics in 2009: 86% male, median age 48 years (IQR 47–66), 86% on cART, median CD4+ T-lymphocytes 532 cells/mm^3^ (IQR 349–725), baseline HIV-1 RNA 71% <20 copy/ml, 14% 20–400 copy/ml, 14% >400 copy/ml. Population characteristics in 2005: 64% on cART, median CD4+ T-lymphocytes 473 cells/mm^3^ (IQR 285–752), baseline HIV-1 RNA 57% <50 copy/ml, 14% 50–400 copy/ml, 29% >400 copy/ml.

## Discussion

In HIV-infected patients on cART, with a median CD4+ T-lymphocyte count above 500 cells/mm^3^, one dose of MF59-adjuvanted 2009 pandemic influenza A(H1N1) vaccine induced a high rate of seroprotection comparable to that in healthy controls. The second dose showed no effect on GMT 5 weeks after it had been administered, but it did have a modest additional effect on the seroprotection rate in HIV-infected patients. Post-vaccination GMT was lower in HIV-infected patients than in healthy controls. Furthermore we found that seasonal TIV induced seroprotection to pH1N1 in just under half of the participants and that this effect was more pronounced in older subjects.

There are three published studies and there is one set of preliminary data on the humoral response to a single dose of 2009 pandemic influenza A(H1N1) vaccine in comparable groups of HIV-infected patients (Table 4) [11]–[14]. This study is the first to report the effect of this particular vaccine in HIV-infected patients and the first to report the effect of a second dose in HIV-infected patients. It is also the only study on pH1N1 vaccine in HIV-infected patients that included a comparator control group. In two of the other studies with ASO3-adjuvanted vaccine, the seroprotection rate exceeds 90%. A third study reports a lower seroprotection rate. In a head to head comparison, squalene based adjuvanted influenza vaccine clearly outperforms unadjuvanted influenza vaccine in HIV-infected patients [11], as has also been found for healthy adults [15]. Due to relatively high baseline HI titers, the seroconversion rate in our study was lower than in other studies. A fourfold increase in titer is more difficult to achieve if the baseline titer is already high. This reasoning is in line with the fact that we found higher seroconversion rates for the participants who had undetectable pH1N1 HI titers at baseline. Our interpretation of the data is that most participants in our study were clinically protected following vaccination with MF59-adjuvanted pH1N1 vaccine.

**Table 4:** Comparison of the immunogenicity of a single dose of 2009 pandemic influenza A(H1N1) vaccine in HIV-infected patients.

Study	Number of HIV-infected patients	Vaccine	Vaccine HA,content, µg	Age in years –mean (SD) or *median (IQR)*	CD4+ T-lymphocytes,cells/mm^3^ -mean (SD) or *median (IQR)*	nadir CD4+ T-lymphocytes, cells/mm^3^ - mean (SD) or *median (IQR)*	cART - (%)	HIV-RNA below detection limit – (%)*	Prior 2009-2010 seasonal TIV - (%)	HI titer ≥1∶40 before pH1N1 vaccination - (%)	HI titer ≥1∶40 after one pH1N1 vaccination - (%)	GMT before vaccination – value(95% CI) or *median (IQR)*	GMT after one accination – value (95% CI) or *median (IQR)*	seroconversion rate – (%)	seroprotection rate – (%)#
Launay et al.^11^	154	ASO3-adjuvanted	3.75	*47* *(39–54)*	*523* *(387–752)*	NA	77	77	NA	7	95	8(7–9)	202(172–236)	92	95
	152	unadjuvanted	15	*47* *(40–54)*	*548* *(422–702)*	NA	78	73	NA	9	77	8(7–9)	128(104–158)	72	77
Orlando et al.^14^	253	ASO3-adjuvanted	3.75	47 (10)	570 (266)	NA	91	82	NA	26	92	*5* *(5–40)*	*160* *(80–320)*	83	92
Bickel et al.^12^	160	ASO3-adjuvanted	3.75	46 (10)	514 (246)	160(134)	90	70	11	14	75	9(8–10)	94(73–122)	69	75
Tebas et al.^13^	120	unadjuvanted	15	*46* *(40–53)*	*502* *(307–640)*	131(37–253)	99	84	NA	25	69	NA	NA	56	71
Ourstudy	57	M59-adjuvanted	7.5	52 (11)	*507* *(349–697)*	*143* *(32–281)*	91	79	89	44	88	23(15–35)	110(81–150)	53	88

*HIV-RNA detection limits vary between the different studies.

# Seroprotection rates pertain to all included subjects, irrespective of baseline HI antibody titers. NA: not available.

In this study just under half of the participants had a HI titer ≥1∶40 at baseline, i.e. at or above the threshold that defines seroprotection. Although the peak incidence of the influenza pandemic in the Netherlands coincided with the start of the vaccination campaign [16], less than 10% had a recent influenza-like illness before receiving the first pH1N1 vaccine. Therefore, it seems unlikely that infection with influenza accounted for the high seroprotection rate at baseline. There was a strong association between recent vaccination with 2009–2010 seasonal TIV and seroprotection at baseline. This association was confirmed by analyses of stored serum samples, which showed that 2009–2010 seasonal TIV induced cross-reactive antibodies to pH1N1 and that 2005–2006 seasonal TIV had a comparable effect. In other studies baseline seroprotection rates vary from 0 to approximately 30% [15], [17]–[27]. Some studies do [17], [20], [26] and others do not [23], [24] report an association between baseline HI titers to pH1N1 and prior vaccination with seasonal TIV. The fact that we found a stronger association between vaccination with seasonal TIV and induction of cross-reactive antibodies to pH1N1 than most other studies can be due to a number of reasons. Firstly, as opposed to most other studies, the majority of subjects in our study had received 2009–2010 seasonal TIV before inclusion. Secondly, the time between having received seasonal influenza vaccine and pH1N1 vaccine was much shorter in our study than in other studies. Lastly, we studied HIV-infected patients and it could be that this group produces larger quantities of cross-reactive antibodies upon vaccination because of a less well regulated B-cell immune response [28], [29].

Using virus neutralization assays, others have shown that cross-reactive antibodies that are induced by seasonal TIV are functional against pH1N1 [30]. This entails that these antibodies do confer protection against pH1N1. There is epidemiological evidence that supports this claim although there is also evidence to the contrary [31]–[34]. The surface hemagglutinin and neuraminidase proteins in recent seasonal trivalent inactivated influenza vaccines are antigenically very distant from those of pH1N1. Therefore seasonal TIV is generally not expected to confer a significant degree of cross-protection to pH1N1 [35]. Only older age by way of exposure to pre-1957 influenza strains has consistently been found to confer a relevant degree of cross-reactive antibodies to pH1N1 [17], [36]–[39]. In this respect it is interesting that we found that seasonal TIV was more likely to induce cross-reactive antibodies to pH1N1 in older than in younger subjects, which contradicts the conclusion of Hancock et al. who found that seasonal TIV induces little to no cross-reactive antibody response to pH1N1 in any age group. We think that our findings show that seasonal influenza vaccines do not induce a relevant degree of cross protection to pH1N1 in (younger) immunologically naïve subjects but that seasonal influenza vaccines can boost relatively unrelated influenza specific memory B-cells. In older individuals who have been exposed to influenza strains or vaccines that are antigenically more related to pH1N1, such boosting induces measurable levels of antibodies to pH1N1, which may augment clinical protection against pH1N1.

This study has strengths and limitations. It was a prospective well controlled cohort study in a fairly homogenous group in which follow-up was complete for 99% of participants. This study is unique in that it shows the immune response to vaccination with pH1N1 and the effect of seasonal influenza vaccines in the same HIV-infected patients. Although symptoms of an influenza-like illness were systematically assessed, respiratory samples were not collected to confirm pH1N1 infection and therefore intercurrent infections can not be excluded. However, only 11 participants had an influenza-like illness between day 0 and day 21. Regarding the generalizability of our results: 91% of our HIV-infected patients were successfully being treated with combination antiretroviral therapy (cART) and very few HIV-infected participants had a CD4+ T-lymphocyte count below 200 cells/mm^3^.

In conclusion, a single dose of MF59-adjuvanted 2009 pandemic influenza A(H1N1) vaccine in HIV-infected patients on cART with a median CD4+ T-lymphocyte count above 500 cells/mm^3^ induced a high rate of seroprotection comparable to that in healthy controls. A second dose had a modest additional effect in HIV-infected patients but not in healthy controls. Post-vaccination GMT, a sensitive marker of the immune competence of a group, was lower in HIV-infected patients than in healthy controls, reflecting the underlying immunodeficiency. Furthermore we found that recent seasonal TIV induced a high rate of age-dependent cross-reactive seroprotection to pH1N1. We think that in general, seasonal TIV boosts pre-existent influenza specific memory B-cells. In older people who in the past have been exposed to influenza strains that are antigenically more alike to pH1N1, this effect induces measurable levels of cross-reactive antibodies to pH1N1. If such an effect is true and if it adds to clinical protection against pH1N1, it is an additional benefit of annual influenza vaccination.
